# Chemical composition and physicochemical properties of natural therapeutic mud of Kazakhstan salt lakes: a review

**DOI:** 10.1007/s10653-023-01813-3

**Published:** 2024-01-16

**Authors:** Khafiza Akimzhanova, Alfira Sabitova, Binur Mussabayeva, Zhaksyntay Kairbekov, Bulbul Bayakhmetova, Jędrzej Proch

**Affiliations:** 1 Department of Chemical Technology and Ecology, Shakarim University, Semey, Kazakhstan; 2 Higher School of Natural Sciences, Astana International University, Astana, Kazakhstan; 3 Department of Chemistry and Chemical Technology, Al Farabi Kazakh National University, Almaty, Kazakhstan; 4 Department of Analytical Chemistry, Faculty of Chemistry, Adam Mickiewicz University, Poznań, Poland

**Keywords:** Natural mud, Peloid, Brine, Silt, Salt lake, Chemical characteristic

## Abstract

In recent years, interest in natural therapeutic mud has been growing all over the world. This natural product has a positive therapeutic effect on the skin and has fewer side effects on the human body. There are more than 40 thousand salt lakes in Kazakhstan. Most of them have natural mud sources, the potential of which has not yet been fully explored. The review presents an analysis of the available information on the physical and chemical properties of the main sources of natural mud from salt lakes in Kazakhstan and Kazakh sanatoriums that use natural mud in the treatment. All available publications, presenting the systematic studies, were used for data analysis. A comparative analysis of the mineralization of water, brine, and silt in one reservoir shows that the mineralization of water is not always the least. The available data indicate a point and partial nature of peloid studies, e.g., inorganic composition of natural muds from Western and Southern Kazakhstan is well described in the literature. In turn, there is a lack of these data from Northern and Eastern Kazakhstan. Studies of peloids in these regions seem to be a promising direction of the future research for both local and world scientists. What is more, there is also a big gap in the analysis of organic matter of muds from the Kazakh lakes. Comparing the state of the art, i.e., the studies from other parts of Asia and Europe, the identification of the organic part of muds is another desirable direction as a potential source of biologically active compounds of natural origin.

## Introduction

Salt lakes of Kazakhstan are gaining popularity every year due to the healthful effect of their water and mud. The composition, properties, preparation, maturation and therapeutic use of peloids have been studied for years; however, there is still not enough information on the chemical composition of peloids in the salt lakes of Kazakhstan. There is also no convincing evidence for a scientific explanation of the therapeutic effects of these natural muds. In this regard, a review of recent research on peloids and pelotherapy in Kazakhstan was conducted in order to systematize the data and summarize information for further research in this area.

According to the definition formulated by the International Society of Medical Hydrology, mud or peloid is a natural product consisting of a mixture of salty lake or mineral healing water (liquid phase) with organic and inorganic components (solid phase) obtained as a result of biological (humus) and geological effects (clay minerals), which is applied locally as a therapeutic agent in the form of applications (Antonelli & Donelli, 2018). All types of peloids can be divided into two classes: natural peloids and peloids sensu strictu. The first class includes mud or slurry, which is ripened under suitable conditions in nature. It may also have such names as fango, loam, sapropel, peat, and biofilm. The second class includes mud or silty suspension that the maturation occurs in spas or research laboratories (where peloids of natural origin undergo further maturation) (Gomes et al., 2013). The conditions and time of maturation can change some characteristics of peloids, such as their plasticity, absorption ability, and biochemical composition (Carretero, 2002; Centini et al., 2015). The formation of the chemical composition and genesis of silt mud are influenced by the salt composition of brine and soil, as well as organic matter of plant and animal origin. The degree of mud accumulation is greatly influenced by the morphological features of reservoirs, the salinity of water, the geological structure of the banks and the associated features of the landscape (Verigo, 1892). The process of mud formation is determined by a complex interaction of geological-hydrological, climatic, physical-chemical and biological factors (Bokuchava, 2009). Natural or anthropogenic origin of heavy metals in sediments depends on the metal and the geographical location (Udayakumar et al., 2014). Since sedimentation is accumulative in nature, studying the elemental composition, especially heavy metals, will make it possible to assess the ecological state of this hydromineral resource and draw conclusions about the safety of its use (Sibin et al., 2022).

There are more than 48 thousand lakes in Kazakhstan, of which about 45 thousand are small lakes with the area less than 1 km^2^ (Muravlev, 1973). The variety of relief and moisture conditions causes uneven distribution of natural lakes. Northern Kazakhstan accounts for 45% of lakes, Central and Southern—36%, and other regions—19%. The water and salt balances of lakes are mainly related to zonal conditions. In accordance with the increase of aridity from north to south, the share of drainless lakes and salinity of lake waters increases to the south. Along with active studies of present and future water availability in Kazakhstan, there is a lack of complete information on the lake state, except for the unique water bodies studied separately: the Caspian Sea, the Aral Sea, Balkhash Lake, etc. (Zhumangalieva, 2014). Similarly, information on deposit reserves for most of the studied mud springs is very limited. The water supply to the lakes mainly occurs from underground and melted snow waters. In the usually arid climate of Southern and Western Kazakhstan in summer, water evaporation increases. Consequently, the concentration of chemical elements dissolved in water increases and a highly mineralized layer of mud forms at the bottom of the reservoir. The mud often ends up on the surface. The concentration and composition of brine are varied depending on meteorological conditions and periods of the year (Tokpanov, 2016). More detailed geological exploration was carried out by several groups of researchers for the lakes of Northern Kazakhstan. The thickness of the mud layer of Lake Krivoe is 30–70 cm, and the estimated reserves are about 1 million m^3^. Almost the entire area of Lake Stanovoe is covered by mineral black, ointment-like silt hydrogen sulfide mud with a thickness of 0.3 m. Lake Solenoe is located in the southeastern part of the bottom of the basin of Lake Stanovoe. Its area is 0.25 km^2^, and mud deposits are 0.239 km^2^. Their thickness increases from the shore to the center of the lake from 0.03 to 0.54 m. The thickness of the mud layer of Lake Minkeser reached 20–30 cm over a large area (in some places more) with a total volume of at least 30000 m^3^. The bottom sediments of Lake Safonkovo are represented by a layer of jelly-like brown sapropel about 25–30 cm thick (Fomin et al., 2012). Bottom silt deposits of the Lake Alakol in Southern Kazakhstan are represented by two main horizons: (1) upper black silt and (2) underlying dark gray silt (underlain by gray clayey silts and sand). The thickness of the black silt layer reaches an average of 0.4 m, and as it approaches the shore, the layer decreases to 5 cm and is replaced by dark gray silts (Tokpanov et al., 2019). Lake Zhalanashkol is part of the Alakol lake system. It has a water surface area of 37.5 km^2^, a coastline length of 23.8 km, a water volume of 104 million m^3^, and a depth of 2.6 m (Tokpanov, 2016). In turn, Lake Rey has an area of about 1.2 km^2^ with a maximum length of 400 m and a width of 150 m, the length of the coastline is 1150 m, the water volume is 156 thousand m^3^, and the average depth is 2.6 m (Tokpanov et al., 2021). Of the lakes of Western Kazakhstan, with the geomorphological and hydrochemical characteristics, only Lake Inder (the Atyrau region) is presented in detail. Most of the salt lakes of Western Kazakhstan, which have reserves of potentially therapeutic mud, have not been studied. Of the six studied salt lakes, five are located in the Caspian lowland (Inder, Alzhansor, Aralsor, Khakisor, Bolshoy Sor), while Lake Sorkol on the Subural Plateau. Three lakes (Bolshoi Sor, Alzhansor, and Sorkol) are located in the dry steppe zone, Lake Aralsor in the semi-desert zone, and two lakes (Khakisor, Inder) in the desert zone. Lake Bolshoy Sor has a round shape with a diameter of about 2.5 km and is covered with a salt crust, under which there is a layer of mud. The size of Lake Alzhansor is 1.5 × 1.5 km, Lake Sorkol 2.1 × 1.6 km and an area of about 750 h, and Lake Khakisor 65–75 × 2–15 km and an area of 300–400 km^2^. The largest of the studied lakes is Lake Aralsor, with an area of 10000 h (Akhmedenov, 2020).

**Fig. 1:**
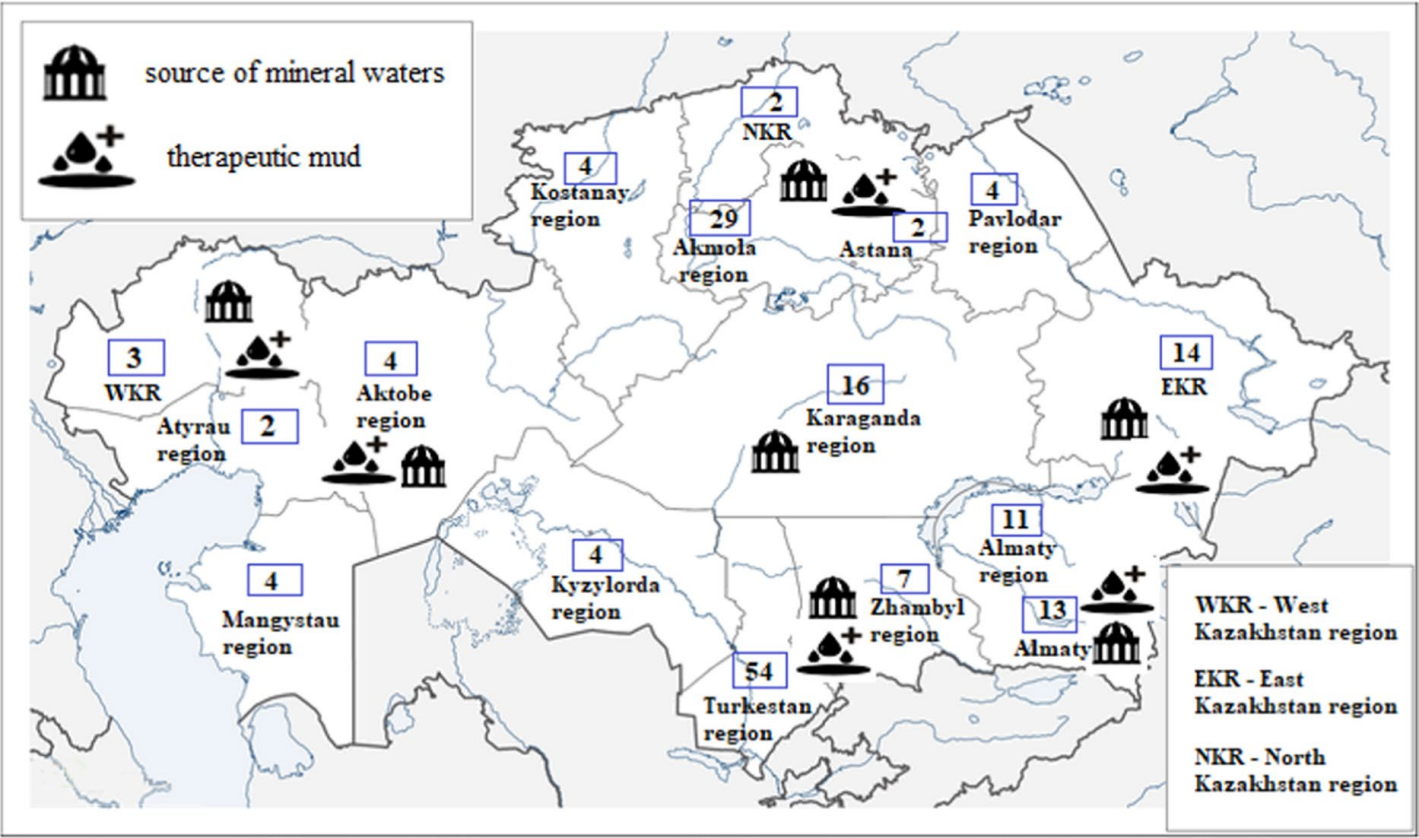
Territorial and quantitative distribution of health balneological resorts in Kazakhstan

There is a National Resort Association in the republic, which carries out sectoral coordination of the subjects of the sanatorium and resort industry. At the beginning of 2021, there were 173 health resorts in Kazakhstan, of which 9 large resorts use therapeutic mud and brine from their own wells (Fig. 1). The rest of the resorts uses imported mud for the treatment (Yessengabylova et al., 2016).

For qualitative assessment of the mineralization of natural lakes, the widely accepted classification is used. According to this, freshwater (*f*), brackish (*b*), saline (*s*) and hypersaline (*h*) lakes are characterized by salinity values *S* ≤ 3.0, 3.0 < *S* ≤ 27.5, 27.5 < *S* ≤35.0, *S* >35.0‰, respectively. At that, lake water with *S*>50‰ can be referred to brine (rapa)—salt-saturated water of saline lakes. Most of the boundaries of categories in the lake classification by salinity are conditional (Williams, 1996).

Depending on the geographical location and climatic conditions, fluctuations in the composition and content of mineral and organic substances of peloids vary quite widely indicating their individual specificity in each case. We believe that there is little scientifically substantiated information in the literature on the relationship of the chemical composition of therapeutic mud of the salt lakes of Kazakhstan with their well-known therapeutic properties, such as a positive effect on joints, skin, respiratory organs, and the consequences of injuries.

## Methodology

Articles related to characteristics of peloids in the Kazakh context were searched through a number of databases such as Scopus (Elsevier), Web of Science (Clarivate), and Google Scholar. The search was done under different topics related to natural mud of Kazakhstan including synonyms of the object to collect data about chemical properties, mineralogy, therapeutic effect, and environmental effects to cover all available information and understand the degree of research on the composition of peloids in Kazakhstan. The search was done with data from 2012 to 2022 and only Kazakhstan country limitations. The literature was selected as follows:

- Keyword “Peloid” – 130 in Scopus; 252 in Web of Science; 20,600 in Google Scholar publications.
- Limit to 2012–2022 – 86 in Scopus, 162 in Web of Science; 11,000 in Google Scholar publications.
- Limit to the keywords “Peloid” AND “Kazakhstan” – 4, of which reviews-2, articles–2 in Scopus; 0—in Web of Science; 6—in Google Scholar publications.

Information was presented on main sources and studied parameters to present the physicochemical characteristics of natural mud in Kazakhstan.

## Results

### Analysis of the main sources of research object

Table 1 presents a generalized overview of the studies of the chemical nature of mud by publications, including the initial data, methods, and procedures used. Among the groups of researchers, there are two scientific communities engaged in research, mainly related to the active study of the tourist and recreational potential of various natural zones of Kazakhstan, including salt lakes and their hydromineral resources.

**Table 1:** General information on natural mud research in Kazakhstan

The object of the study	Region	Timeframe	Research methods	References
Water and mud of Zhalanashkol Lake	South Kazakhstan	2012–2015	Atomic absorption spectrometry (Hitachi 180–50, Japan), flame photometry (PFP7, UK, Inductively coupled plasma optical emission spectrometry (Optima 2000 DV, USA)	Tokpanov (2016)
Mud of “Kossor” deposit on the southern shore of Alakol Lake	South Kazakhstan	Not specified	Atomic absorption spectrometry (Hitachi 180–50, Japan), flame photometry (PFP7, UK, inductively coupled plasma optical emission spectrometry (Optima 2000 DV, USA)	Dzhetimov et al. (2014)
				Tokpanov et al. (2019)
Water and mud of Ray Lake	South Kazakhstan	2012–2015	Atomic absorption spectrometry (Hitachi 180–50, Japan), flame photometry (PFP7, UK, inductively coupled plasma optical emission spectrometry (Optima 2000 DV, USA)	Tokpanov et al. (2021)
Muds of lakes: Zhaman, Krivoye, Stanovoe, Kisloe, Minkeser, Safonkovo	North Kazakhstan	Not specified, except Stanovoe Lake-1962	Not specified	Fomin et al. (2012)
Muds of lakes: Khakisor, Inder, Aralsor, Alzhansor, Bolshoy Sor, Sorkol	West Kazakhstan	2017–2020	Atomic absorption spectrophotometry (Varian AA-140); Spectrophotometry	Akhmedenov (2018)
				Akhmedenov (2020)
				Myazina (2019)
				Khalelova et al. (2020)
				Akhmedenov and Khalelova (2021)
				Khalelova and Kalyuzhnaya (2022)

The first community includes a team of researchers under the leadership of Tokpanov, engaged in the study of water resources of South Kazakhstan. The research was conducted from 2012 to 2015. Implementing the research project, 40 water samples were taken from different depths from 15 points at a distance of 120 m from each other, and 20 samples of therapeutic mud in the case of Zhalanashkol Lake; 30 water samples taken from 10 points at a distance of 120 m from each other; and 13 samples of natural mud in the case of Ray Lake in accordance with ST RK GOST P 51592-2003 (Table 1).

There is another group of scientists led by K.M. Akhmedenov, who have conducted several studies on the properties of peloids from saline lakes in Western Kazakhstan (Table 1). Samples were taken at a depth of 0.3–0.5 m in an amount of 1 l. Sampling was carried out according to generally accepted methods with fixation in place: (a) reservoir parameters and sampling conditions (reservoir name, air and water temperature, date and place/coordinates of sampling, sampling depth, silt deposit capacity, and brine layer height above the mud deposit surface); (b) organoleptic characteristics of mud samples (color, odor, consistency, and structure); (c) the presence and nature of inclusions (salt crystals, sand, and plant residues). Similar research data on peloids of Alzhansor and Aralsor lakes were also presented by Myazina (2019). In the published studies conducted by Tokpanov et al. and Akhmedenov et al., the methods and procedures are well described that characterizes the validity of their results (Table 1). For mud of the northern region, there are only visual descriptions of therapeutic mud deposits (Fomin et al., 2012; Kan et al., 2019). These studies have a review character without presenting the methodology of the research.

The data obtained from the literature analysis indicate that in different years, the natural mud of the salt lakes of Southern Kazakhstan, Western Kazakhstan, and Northern Kazakhstan was studied in a greater extent. Information on research of natural muds of the northeastern region is not available in the public domain. Thus, the small number of publications and their introductory and advertising nature demonstrate the insufficiency of research in this area. According to the lack of scientific data in North-Eastern Kazakhstan, it is a desirable direction of the future research, both for local and world researchers. Moreover, the increasing demand for the use of natural mud among the population emphasizes the relevance of research.

### Physicochemical characteristics of the natural peloids of Kazakhstan

Based on all available data, a general overview of the organoleptic properties of mud is presented in Table 2. The most saturated and oversaturated mud occurs in the Inder, Aralsor, Krivoe, and Stanovoe lakes, and the mineralization of mud solution is 237.8–377 g/L.

**Table 2:** Classification and organoleptic properties of the studied peloids

The object of the study	Region	Organoleptic properties of natural mud	Type of mud	References
Mud of “Kossor” deposit on the southern shore of Alakol Lake	South Kazakhstan	Plastic greasy colloidal homogeneous mass of black color with a slight smell of hydrogen sulfide	Sulfide silt	Djetimov et al. (2014)
				Tokpanov et al. (2019)
Mud of Inder lake	West Kazakhstan	Black dense consistency and heterogeneous structure, with the smell of wet bitumen	Strongly sulfide, weakly alkaline	Akhmedenov (2020)
				Akhmedenov and Khalelova (2021)
Mud of Alzhansor lake	West Kazakhstan	Soft, plastic mass with a faint smell of bitumen with a dense consistency and homogeneous structure	Strong sulfide, slightly alkaline	Akhmedenov (2020)
				Akhmedenov and Khalelova (2021)
Mud of Khakisor lake	West Kazakhstan	Brown, thick consistency, lumped, homogeneous structure, odorless	Sulfide, slightly alkaline	Akhmedenov, 2020;
				Akhmedenov and Khalelova (2021)
Mud of Aralsor lake	West Kazakhstan	Light gray, thick creamy consistency, heterogeneous structure, odorless	Sulfide, slightly alkaline	Akhmedenov (2020)
				Akhmedenov and Khalelova (2021)
Mud of Sorkol lake	West Kazakhstan	Light gray color, thick consistency, plastic, with inclusions of sand and stones, odorless	Slightly sulfide, slightly alkaline	Akhmedenov (2020)
				Khalelova et al. (2020)
				Akhmedenov and Khalelova (2021)
				Khalelova and Kalyuzhnaya (2022)
Mud of Bolshoy Sor lake	West Kazakhstan	Gray, thick consistency, plastic, without large inclusions, odorless	Slightly sulfide, highly mineralized	Akhmedenov (2020)
				Khalelova et al. (2020)
				Akhmedenov and Khalelova (2021)
				Khalelova and Kalyuzhnaya (2022)
Mud of Minkeser lake	North Kazakhstan	Not specified	Sulfide-silt	Fomin et al. (2012)
Mud of Kisloe lake	North Kazakhstan	Black and dark gray soft-plastic, slightly clogged with sand and plant residues with the smell of hydrogen sulfide	Silt	Fomin et al. (2012)
Mud of Stanovoe lake	North Kazakhstan	Homogeneous, viscous fluid consistency with the smell of hydrogen sulfide	Not specified	Fomin et al. (2012)

**Fig. 2:**
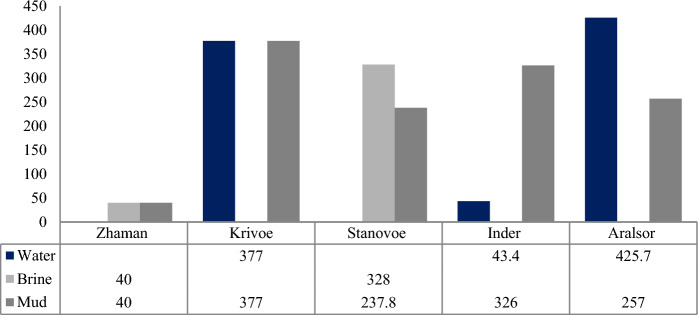
Mineralization of water, brine, mud of lakes of Kazakhstan, g/L **(**Akhmedenov & Khalelova, 2021; Fomin et al., 2012)

Comparing the mineralization of water and brine of the same lakes, the different relations are observed. In the case of Lake Inder, the mineralization of water is 7.7 times less in comparison with mud, while the mineralization of water and brine is 1.6 and 1.4 times more in the case of lakes Aralsor and Stanovoe, respectively. In the case of Lake Krivoe and Zhaman, this indicator coincides with water and mud (Fig. 2). Summarizing these data, a natural process may be that the salt content is not always highest in the mud.

Summary information about the content of inorganic components in Table 3 and physicochemical parameters of peloids are presented in Table 4, The mud of lakes of Western Kazakhstan, such as Inder and Alzhansor, belongs to highly sulfide mud (from 550 to 560 mg/100 g of sample); the rest of the studied peloids are medium sulfide (from 150 to 470 mg/100 g of sample). The mud of Sorkol (Western Kazakhstan) and Stanovoe (Northern Kazakhstan) lakes is weakly sulfide. The majority of considered peloids have slightly alkaline medium (pH = 7.0–9.2), except for Solyonoye and Minkeser lakes with slightly acidic environment. Analysis of the composition of the studied peloids shows that most of them belong to highly mineralized (35–150 g/L), saturated (150–300 g/L), medium sulfide (0.15–0.5% of natural mud), and slightly alkaline mud (7.0–9.0) of continental origin. An analysis of the available data on the microaggregate composition shows that the low content of the sandy fraction of the peloids of lakes Alzhansor, Khakisor, Big Sor of Western Kazakhstan, lakes Kisloye and Stanovoye of Northern Kazakhstan, as well as the high content of the fine clay fraction of the mud of the Kossor deposit of Southern Kazakhstan indicates their long-term maturation. (Bergamaschi et al., 2020). In terms of the quantitative composition of the clay fraction, the peloids of Northern Kazakhstan are similar to the peloids of Sečovlje Salina Piran Bay, Slovenia (Glavaš et al., 2016).

**Table 3:** Inorganic composition of natural mud of salt lakes of Kazakhstan

The object of the study	Region	Total concentration of elements, mg/100 g WW	Concentration of water-soluble ions, mg/100 g WW	Inorganic matter content, mg/L of mud solution	References
Mud of “Kossor” deposit on the southern shore of Alakol lake	South Kazakhstan	ND	H_2_S-140	ND	Djetimov et al. (2014)
					Tokpanov et al. (2019)
Mud of Zhalanashkol lake	South Kazakhstan	ND	Na^+^-30200; K^+^-20; Ca^2+^-210; Mg^2+^-180; Cl^−^-190; SO_4_^2−^-62380	ND	Tokpanov (2016)
Mud of Ray lake	South Kazakhstan	Cu-0.46; Ni-1.13; Pb-0.30; Cd-0.004; Mn-1.60; As-0.81; Zn-0.97; Cr-0.002	ND	ND	Tokpanov et al. (2021)
Mud of Inder lake	West Kazakhstan	FeS–550; Zn-3.15; Cu-1.00; Pb-4.97; Cd-0.18; Mn-36.4;	H_2_S-300; Ca^2+^-3200; Mg^2+^-1200; Cl^−^-33015; SO_4_^2−^-5760;	Br^−^-40.80; I^−^-1.8	Akhmedenov (2020); Akhmedenov and Khalelova (2021); Myazina (2019)*
Mud of Alzhansor lake	West Kazakhstan	FeS–560; Zn–2.69; Cu–0.89; Pb–3.87; Cd–0.13; Mn–88.9;	H_2_S–100; Ca^2+^-1600; Mg^2+^-6480; Cl^−^-31240; SO_4_^2−^-10560;	Br^−^-104.3; I^−^-0.1; H_3_BO_3_–8.1	Myazina (2019)*; Akhmedenov (2020)**; Akhmedenov and Khalelova (2021); Khalelova and Kalyuzhnaya (2022)
Mud of Khakisor lake	West Kazakhstan	FeS–150; Zn–2.63; Cu–0.77; Pb–0.01; Cd–0.30; Mn–76.9	H_2_S–10;	Br^−^-120.8; I^−^-0.1; H_3_BO_3_–8.9	Akhmedenov (2020); Akhmedenov and Khalelova (2021)
Mud of Aralsor lake	West Kazakhstan	FeS–470; Zn–2.50; Cu–0.75; Pb–0.71; Cd–0.28; Mn–36.0;	H_2_S–30; Ca^2+^-800; Mg^2+^-3600; Cl^−^-30,175; SO_4_^2−^-9600;	Br^−^-87.9; I^−^-0.7	Akhmedenov (2020); Akhmedenov and Khalelova (2021); Myazina (2019)
Mud of Sorkol Lake	West Kazakhstan	FeS–110; Zn–8.02; Cu–0.25; Pb–1.24; Cd–0.65; Mn–24.0;	H_2_S–20; Na^+^ 22080; Cl^−^-19525; SO_4_^2−^-42240;	Br^−^-139.4; I^−^-0.1; H_3_BO_3_–23.6	Akhmedenov (2020); Khalelova et al. (2020); Akhmedenov and Khalelova (2021); Khalelova and Kalyuzhnaya (2022)
Mud of Bolshoy Sor lake	West Kazakhstan	FeS–320; Zn–4.28; Cu–1.52; Pb–1.55; Cd–0.13; Mn–37.8;	H_2_S–40; Na^+^-14260; Ca^2+^-5600; Mg^2+^-5520; Cl^−^-34435;	Br^−^-123.8; I^−^-0.1; H_3_BO_3_–3.0	Akhmedenov (2020); Khalelova et al. (2020); Akhmedenov and Khalelova (2021); Khalelova and Kalyuzhnaya (2022)
Mud of Minkeser lake	North Kazakhstan	FeO-250	H_2_S–29	ND	Fomin et al. (2012)
Mud of Kisloe lake	North Kazakhstan	ND	H_2_S–70	ND	Fomin et al. (2012)
Mud of Stanovoe Lake	North Kazakhstan	FeS–140; SiO_2_–230; A1_2_O_3_–1220; Fe_2_O_3_–1300; MnO–30;	Mg^2+^–624; Cl^−^-3160; SO_4_^2−^–960;	H_3_BO_3_–70;	Fomin et al. (2012)

WW–wet weight, ND–no data

**Table 4:** Physicochemical parameters of natural mud of salt lakes of Kazakhstan

The object of the study	Region	Physical parameters				Chemical parameters			References
		Humidity, %	Redox potential, mV	Heat capacity, cal/g⋅°C	Microaggregate composition, mm—%	pH	Mineralization, g/L of mud solution	Organic matter content, g/100 g WW	
Norm for sulfide silt mud, units of measurement		25–75	− 500–0	0.4–0.9	> 0.25-no more than 3.0; > 0.5–no	7.0–9.0	1-over 150	not less than 0.5	Adilov et al. (2000)
Mud of “Kossor” deposit on the southern shore of Alakol lake	South Kazakhstan	42.9	ND	0.847	> 0.001–44; > 0.25–0.3; > 0.5–absent	9.2	ND	ND	Djetimov et al. (2014); Tokpanov et al. (2019)
Mud of Zhalanashkol lake	South Kazakhstan	ND	ND	ND	ND	ND	ND	ND	Tokpanov (2016)
Mud of Ray lake	South Kazakhstan	ND	ND	ND	ND	ND	ND	ND	Tokpanov et al. (2021)
Mud of Inder lake	West Kazakhstan	26.1	263	0.76	> 0.001–ND; > 0.25–2.99; > 0.5 –absent	7.0; 7.1*	73*; 326	1.07	Akhmedenov, (2020); Akhmedenov and Khalelova (2021); Myazina (2019)*
Mud of Alzhansor lake	West Kazakhstan	47.3	310	0.579; 0.840**	> 0.001–ND; > 0.25–1.40; > 0.5–Sand. plant residues	7.6*; 8.0	150*; 152	2.66	Myazina (2019)*; Akhmedenov (2020)**; Akhmedenov and Khalelova (2021); Khalelova and Kalyuzhnaya (2022)
Mud of Aralsor lake	West Kazakhstan	35.2	231	0.82	> 0.001–ND; > 0.25–6.45; > 0.5–absent	7.9*; 8.4	168*; 257	0.92	Akhmedenov (2020); Akhmedenov and Khalelova (2021); Myazina (2019)*
Mud of Khakisor lake	West Kazakhstan	23.1	148 + 112*	0.69*; 0.385	> 0.001–ND; > 0.25–0.78; > 0.5–absent	7.2	160	0.19	Akhmedenov (2020); Akhmedenov and Khalelova (2021)*
Mud of Sorkol lake	West Kazakhstan	27.9	394	0.79; 0.423*	> 0.001–ND; > 0.25–20.7; > 0.5–Sand. plant residues	8.4*; 9.0	195	2.19	Akhmedenov (2020); Khalelova et al. (2020); Akhmedenov and Khalelova,(2021)*; Khalelova and Kalyuzhnaya (2022)
Mud of Bolshoy Sor lake	West Kazakhstan	40.1	214; 268*	0.85; 0.521*	> 0.001–ND; > 0.25–1.06; > 0.5–absent	7.1*; 7.6	124	3.49	Akhmedenov (2020); Khalelova et al. (2020); Akhmedenov and Khalelova (2021)*; Khalelova and Kalyuzhnaya (2022)
Mud of Zhaman lake	North Kazakhstan	65–70	ND	ND	ND	ND	40	ND	Fomin et al. (2012)
Mud of Krivoe lake	North Kazakhstan	ND	ND	ND	ND	ND	377	ND	Fomin et al. (2012)
Mud of Stanovoe lake	North Kazakhstan	ND	− 153	ND	> 0.001–3.2; > 0.25–1.41; > 0.5–absent	7.3	238	1.41, including C–0.82	Fomin et al., 2012
Mud of Kisloe lake	North Kazakhstan	54–73	− 309	0.71	> 0.001–ND; > 0.25–0.32; > 0.5–ND	ND	ND	ND	Fomin et al., 2012
Mud of Minkeser lake	North Kazakhstan	32	ND	0.458	> 0.001–2.35; > 0.25–ND; > 0.5–ND	6.45	ND	ND	Fomin et al., 2012

WW–wet weight, ND–no data; *, **–indication of the references

The content of sulfides in natural muds as well as the pH varies within a wide range regardless of the region. In the mud of the deposit “Kossor” (South Kazakhstan), the mineral bischofite (MgCl_2_) was isolated (Djetimov et al., 2014), which has anti-inflammatory, regenerating and analgesic effect. Comparing the cationic and anionic composition of water and brine, the highest content of magnesium ions in the salt lakes of Northern Kazakhstan was found in comparison with other regions. In turn, generally accepted biologically active components (Adilov et al., 2000) were determined on the higher level in water and brine than in the peloids of the studied samples.

The content of heavy metals is below the standard values adopted by the US EPA (EPA, 2012). A comparative analysis of the composition of heavy metals shows that the peloids of Western Kazakhstan have mostly similar content of manganese, zinc, copper, lead, and cadmium with sapropels from Romanian saline lakes (Baricz et al., 2021), as well as zinc and lead with peloids from Ulcinj coast, Montenegro (Potpara et al., 2017), and San Diego de los Baños Thermal Center, Cuba (Suárez Muñoz et al., 2015). Comparing the available data from other countries, the mud of Southern and Western Kazakhstan has a low copper content; however, it has a high cadmium content. This is most likely explained by the geological characteristics of the regional geophone (Calin et al., 2020).

**Fig. 3:**
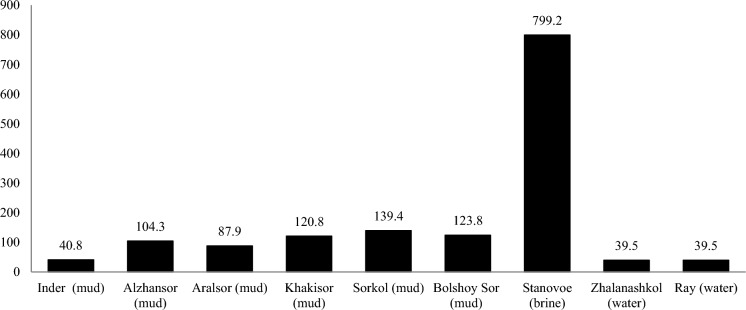
Bromine content in water, brine, mud of lakes in Kazakhstan, mg/L (Akhmedenov, 2020; Akhmedenov & Khalelova, 2021; Fomin et al., 2012; Tokpanov, 2016; Tokpanov et al., 2021)

Boron (in the form of orthoboric acid) is contained below the norm in all the samples studied with the exception of the mud of Lake Stanovoe, where the content of this component was 2 times higher than the lower threshold acceptable for mineral waters, i.e., H_3_BO_3_ = 35 mg/L (Adilov et al., 2000). Metasilicic acid and radon were not detected in the samples. Figure 3 shows a comparative diagram of the content of bromine, which data were determined for most samples of water, brine, and mud. In all investigated samples, the quantity of bromine exceeds the minimal threshold acceptable for mineral waters, i.e., Br = 25 mg/L (Adilov et al., 2000) by 1.6 times in water (Zhalanashkol, Ray Lakes; Southern Kazakhstan); by 32 times in brine of Stanovoe Lake (Northern Kazakhstan); and up to 7.4 times in the mud of lakes Solyonoye, Sorkol, Bolshoy Sor, and Khakisor (Western Kazakhstan). It is worth mentioning that the increased content of bromine has a sedative effect, normalizing the basic nervous processes. In most samples, iodine was found at insignificant amount in comparison with the minimum required for mineral waters, i.e., I = 5 mg/L (Adilov et al., 2000), except the brine of Stanovoe Lake.

A summary analysis of the physical parameters of the mud shows that the moisture, redox potential, and heat capacity of all the muds studied are within the normative values, indicating optimal sulfate-reducing properties (especially for Solyonoye, Alzhansor, Sorkol, Kisloe mud), heat retention properties, and consistency. The content of heavy metals is below the background concentrations (Akhmedenov, 2020; Akhmedenov & Khalelova, 2021).

In general, the data analysis shows the lack of research on the organic part of the mud, which contains a large number of biologically active components, affecting anti-inflammatory properties, having immunostimulating, antitumor and detoxifying action (Carretero, 2020; Centini et al., 2015; Elkayam et al., 2000). Research into the communities of microorganisms in peloids that produce secondary metabolites has made it possible to identify a wide variety of biologically active substances, such as polyketides, peptides, sulfoglycolipids, glycoglycerolipids, etc. The study of their unique structure and composition allows to interpret the positive therapeutic effects of peloids, and it also contributes to research their selective extraction to obtain natural healing concentrates (Vadlja et al., 2022). According to available data, the highest content of organic matter per carbon was determined in the mud of Bolshoy Sor and Alzhansor lakes. Unfortunately, there is no information about this content in muds of other areas as well as in other samples of water and brine.

In the world research, there are examples of the characterization of some organic matters of peloids. In Mongolia, the organic matters of peloids from 12 lakes were investigated using several analytical techniques, e.g., ^13^C NMR spectroscopy, infrared spectroscopy, and gas chromatography–mass spectrometry (GC–MS). This study indicated that the balneological value of peloids was increased by the presence of known bioactive organic compounds, i.e., lipids, carbohydrates, and humic acids as well as hydrogen sulfide (Tserenpil et al., 2010). In Latvia, gyttja are organic-rich freshwater sediments (formed from the remains of plankton, water plants, and benthic organisms) and valuable natural resources used in balneology. Sediments were identified as peaty, green algae, various algae, diatom, organic-silicate, and carbonate types of gyttja. Total content of elements was variable, e.g., the macroelement content was higher for carbonate gyttja, while the microelement content was higher for organic-silicate gyttja. Authors recommended gyttja as a potential peloid in health care after the estimation of the element bioavailability (Vincevica-Gaile & Stankevica, 2018). Along the Croatian coast, organic-rich sediments have been investigated and classified as healing mud, i.e., peloids. However, the total and extractable content of elements must be determined if the sediments are to be used as healing mud (Miko et al., 2007).

## Conclusion

The results of publications presenting studies of peloids of Western and Southern Kazakhstan have been reviewed. On the one hand, investigations concerning hydromineral resources of six large lakes in Western Kazakhstan are distinguished by more extensive research of composition and properties of mud. On the other hand, publications concerning the peloids of South Kazakhstan have a narrower scope focusing on the study of water and brine of Rey and Zhalanashkol lakes. In turn, the available studies of peloids from the northern and eastern regions have either a review character or introductory notes (presented on the official websites of large sanatoriums). According to this, there is observed a large disproportion due to the region of research as well as the lack of comprehensive mud analyzes in a specific region. Most natural muds of Kazakhstan refer to highly mineralized and saturated medium-sulfide weakly alkaline silt of continental origin, have a dense consistency without large inclusions, and have a color from light gray to black. Mineralization, pH, sulfide content, and other parameters can vary significantly within one region, and it may be similar to samples from different regions. The individual character of each mud source affects the absence of direct patterns between the physical and chemical properties of the mud and the general climatic zones. The analysis of these indicators also shows that the mineralization of water is not always less than the mineralization of brine and mud. According to this, there may be other factors contributing to the migration of soluble salts from the mud into the cover water (in addition to the processes of sedimentation and weathering). Moreover, the peloids of the northern region have a high content of magnesium ions, while the content of heavy metals is below the background concentrations in the samples of Western and Southern regions. In turn, the studied mud of Western Kazakhstan is bromine-rich (up to 7.4 times higher than the minimum recommended values for balneological needs). Unfortunately, there is a lack of data on the organic part of the therapeutic mud of saline lakes of Kazakhstan, which may contain significant amounts of biologically active components and thus reveal its therapeutic effect. Comparing the studies from other parts of Asia and Europe, the identification of organic compounds is a desirable direction for both local and world scientists. The deficiency of systematic scientific research of composition and properties of natural mud of lakes in Kazakhstan (especially the northeastern region) indicates the relevance of their further study in order to disclose the full potential of this natural and available raw material.

## Future prospects

More research needs to be done on the muds of the North-Eastern region of Kazakhstan, which also contains large reserves of muds, popular for their healing properties among the local population. To unlock the full potential, it is recommended to investigate the organic substances contained in the mud, which can also have a biologically active effect.
